# 2-Methyl-4-trifluoro­meth­yl-1,3-thia­zole-5-carboxylic acid

**DOI:** 10.1107/S1600536809037325

**Published:** 2009-09-19

**Authors:** Gui-xiang Quan, Ai-lan Luo, Wei-hua Cheng, Jia-ying Xu

**Affiliations:** a The Experiment Center of Chemical Engineering, College of Chemical and Biological Engineering, Yancheng Institute of Technology, Yinbing Road No. 9 Yancheng, Yancheng 224051, People’s Republic of China; b Department of Applied Chemistry, College of Chemical and Biological Engineering, Yancheng Institute of Technology, Yinbing Road No. 9 Yancheng, Yancheng 224051, People’s Republic of China; c Department of Chemical Engineering, Yancheng College of Textile Technology, Liberation Road S. No.265 Yancheng, Yancheng 224005, People’s Republic of China

## Abstract

In crystal of the title compound, C_6_H_4_F_3_NO_2_S, mol­ecules are linked by O—H⋯N and C—H⋯O hydrogen bonds, forming chains.

## Related literature

For a related compound, see: Liu (2004 ▶). For reference structural data, see: Allen *et al.* (1987 ▶).
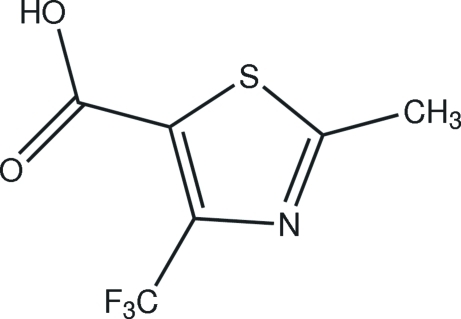


## Experimental

### 

#### Crystal data

C_6_H_4_F_3_NO_2_S
*M* *_r_* = 211.16
Monoclinic, 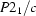

*a* = 4.961 (1) Å
*b* = 15.682 (3) Å
*c* = 10.632 (2) Å
β = 90.35 (3)°
*V* = 827.1 (3) Å^3^
*Z* = 4
Mo *K*α radiation
μ = 0.41 mm^−1^
*T* = 293 K
0.30 × 0.20 × 0.10 mm

#### Data collection

Enraf–Nonius CAD-4 diffractometer
Absorption correction: ψ scan (North *et al.*, 1968 ▶) *T*_min_ = 0.888, *T*_max_ = 0.960
1672 measured reflections
1494 independent reflections
1209 reflections with *I* > 2σ(*I*)
*R*_int_ = 0.018
3 standard reflections every 200 reflections intensity decay: 1%

#### Refinement

*R*[*F* ^2^ > 2σ(*F* ^2^)] = 0.048
*wR*(*F* ^2^) = 0.158
*S* = 1.15
1494 reflections
119 parameters
H-atom parameters constrained
Δρ_max_ = 0.33 e Å^−3^
Δρ_min_ = −0.25 e Å^−3^

### 

Data collection: *CAD-4 EXPRESS* (Enraf–Nonius, 1994 ▶); cell refinement: *CAD-4 EXPRESS*; data reduction: *XCAD4* (Harms & Wocadlo, 1995 ▶); program(s) used to solve structure: *SHELXS97* (Sheldrick, 2008 ▶); program(s) used to refine structure: *SHELXL97* (Sheldrick, 2008 ▶); molecular graphics: *SHELXTL* (Sheldrick, 2008 ▶); software used to prepare material for publication: *PLATON* (Spek, 2009 ▶).

## Supplementary Material

Crystal structure: contains datablocks global, I. DOI: 10.1107/S1600536809037325/hb5094sup1.cif

Structure factors: contains datablocks I. DOI: 10.1107/S1600536809037325/hb5094Isup2.hkl

Additional supplementary materials:  crystallographic information; 3D view; checkCIF report

## Figures and Tables

**Table 1:** Hydrogen-bond geometry (Å, °)

*D*—H⋯*A*	*D*—H	H⋯*A*	*D*⋯*A*	*D*—H⋯*A*
O2—H2*A*⋯N^i^	0.82	2.02	2.820 (3)	166
C1—H1*A*⋯O1^ii^	0.96	2.34	3.277 (4)	166

Symmetry codes: (i) 
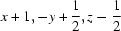
; (ii) 
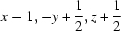
.

**Fig. 1.**
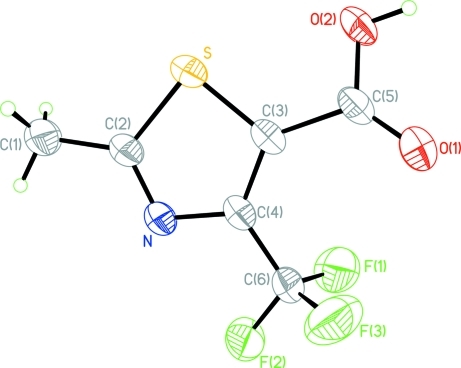
The molecular structure of (I), with displacement ellipsoids drawn at the 50% probability level.

**Fig. 2.**
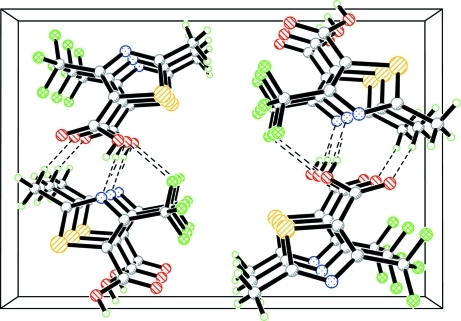
A packing diagram for (I). C—H···O hydrogen bonds are shown by dashed lines.

## supplementary crystallographic information

### Experimental

To a cooled solution of methyl 2-methyl-4-(trifluoromethyl)thiazole-5-carboxylate (0.12 mol) in ethyl alcohol (200 ml) was added a solution of sodium hydroxide (9.62 g) in 200ml of water. The solution was heated at 358 K for 1.5 h. After evaporation of the ethyl alcohol, the aqueous solution was diluted with 200 ml of water and acidified to pH = 1 with concentrated aqueous hydrochloric acid. The solid material was filtered and washed twice with 100 ml of water and 100 ml of dichloromethane. After drying in a vacuum oven,the title compound was obtained  (yield; 85%). Colourless blocks of (I) were obtained by slow evaporation of an ethyl acetate solution.

### Refinement

The H atoms were positioned geometrically (C—H = 0.93–0.96 Å, O—H = 0.82Å) and refined as with *U*_iso_(H) = 1.2*U*_eq_(C) or 1.5U_eq_(methyl C, O).

### Crystal data

**Table:**

C_6_H_4_F_3_NO_2_S	*F*(000) = 424
*M**_r_* = 211.16	*D*_x_ = 1.696 Mg m^−^^3^
Monoclinic, *P*2_1_/*c*	Mo *K*α radiation, λ = 0.71073 Å
Hall symbol: -P 2ybc	Cell parameters from 25 reflections
*a* = 4.961 (1) Å	θ = 10–14°
*b* = 15.682 (3) Å	µ = 0.41 mm^−^^1^
*c* = 10.632 (2) Å	*T* = 293 K
β = 90.35 (3)°	Block, colourless
*V* = 827.1 (3) Å^3^	0.30 × 0.20 × 0.10 mm
*Z* = 4	

### Data collection

**Table:**

Enraf–Nonius CAD-4 diffractometer	1209 reflections with *I* > 2σ(*I*)
Radiation source: fine-focus sealed tube	*R*_int_ = 0.018
graphite	θ_max_ = 25.3°, θ_min_ = 2.3°
ω/2θ scans	*h* = 0→5
Absorption correction: ψ scan (North *et al.*, 1968)	*k* = 0→18
*T*_min_ = 0.888, *T*_max_ = 0.960	*l* = −12→12
1672 measured reflections	3 standard reflections every 200 reflections
1494 independent reflections	intensity decay: 1%

### Refinement

**Table:**

Refinement on *F*^2^	Secondary atom site location: difference Fourier map
Least-squares matrix: full	Hydrogen site location: inferred from neighbouring sites
*R*[*F*^2^ > 2σ(*F*^2^)] = 0.048	H-atom parameters constrained
*wR*(*F*^2^) = 0.158	*w* = 1/[σ^2^(*F*_o_^2^) + (0.1*P*)^2^] where *P* = (*F*_o_^2^ + 2*F*_c_^2^)/3
*S* = 1.15	(Δ/σ)_max_ < 0.001
1494 reflections	Δρ_max_ = 0.33 e Å^−^^3^
119 parameters	Δρ_min_ = −0.25 e Å^−^^3^
0 restraints	Extinction correction: *SHELXL97* (Sheldrick, 2008), Fc^*^=kFc[1+0.001xFc^2^λ^3^/sin(2θ)]^-1/4^
Primary atom site location: structure-invariant direct methods	Extinction coefficient: 0.050 (8)

### Special details

**Table:**

Geometry. All esds (except the esd in the dihedral angle between two l.s. planes) are estimated using the full covariance matrix. The cell esds are taken into account individually in the estimation of esds in distances, angles and torsion angles; correlations between esds in cell parameters are only used when they are defined by crystal symmetry. An approximate (isotropic) treatment of cell esds is used for estimating esds involving l.s. planes.
Refinement. Refinement of F^2^ against ALL reflections. The weighted R-factor wR and goodness of fit S are based on F^2^, conventional R-factors R are based on F, with F set to zero for negative F^2^. The threshold expression of F^2^ > 2sigma(F^2^) is used only for calculating R-factors(gt) etc. and is not relevant to the choice of reflections for refinement. R-factors based on F^2^ are statistically about twice as large as those based on F, and R- factors based on ALL data will be even larger.

### Fractional atomic coordinates and isotropic or equivalent isotropic displacement parameters (Å^2^)

**Table:**

	*x*	*y*	*z*	*U*_iso_*/*U*_eq_	
S	1.03912 (15)	0.13345 (4)	0.21544 (7)	0.0482 (4)	
N	0.7571 (4)	0.22213 (14)	0.3665 (2)	0.0406 (6)	
F1	1.1047 (4)	0.41020 (12)	0.3353 (2)	0.0813 (7)	
F2	0.7272 (5)	0.38613 (12)	0.4214 (2)	0.0844 (8)	
F3	0.7499 (4)	0.41298 (12)	0.2261 (2)	0.0816 (7)	
O1	1.2183 (5)	0.35504 (14)	0.0770 (2)	0.0683 (7)	
O2	1.4044 (4)	0.22652 (13)	0.0592 (2)	0.0647 (7)	
H2A	1.4972	0.2497	0.0056	0.097*	
C1	0.6926 (6)	0.06626 (19)	0.3979 (3)	0.0569 (8)	
H1A	0.5699	0.0842	0.4621	0.085*	
H1B	0.8346	0.0330	0.4351	0.085*	
H1C	0.5978	0.0324	0.3368	0.085*	
C2	0.8109 (6)	0.14311 (16)	0.3348 (3)	0.0426 (7)	
C3	1.0636 (5)	0.24181 (18)	0.2054 (2)	0.0401 (7)	
C4	0.8996 (5)	0.27820 (16)	0.2937 (2)	0.0381 (6)	
C5	1.2368 (5)	0.28263 (18)	0.1076 (2)	0.0431 (7)	
C6	0.8700 (6)	0.37191 (17)	0.3191 (3)	0.0465 (7)	

### Atomic displacement parameters (Å^2^)

**Table:**

	*U*^11^	*U*^22^	*U*^33^	*U*^12^	*U*^13^	*U*^23^
S	0.0564 (5)	0.0417 (5)	0.0467 (5)	0.0035 (3)	0.0250 (4)	−0.0018 (3)
N	0.0444 (13)	0.0400 (12)	0.0374 (12)	0.0008 (9)	0.0163 (10)	0.0033 (9)
F1	0.0727 (14)	0.0560 (12)	0.1153 (19)	−0.0172 (10)	0.0111 (12)	−0.0146 (11)
F2	0.1252 (18)	0.0513 (11)	0.0775 (15)	−0.0002 (10)	0.0667 (13)	−0.0088 (9)
F3	0.1088 (19)	0.0586 (12)	0.0774 (15)	0.0297 (11)	0.0010 (13)	0.0105 (10)
O1	0.0765 (17)	0.0537 (14)	0.0752 (17)	0.0031 (11)	0.0441 (13)	0.0126 (11)
O2	0.0737 (15)	0.0559 (13)	0.0651 (15)	0.0091 (10)	0.0492 (12)	0.0065 (10)
C1	0.068 (2)	0.0435 (16)	0.0600 (19)	−0.0010 (14)	0.0290 (16)	0.0046 (13)
C2	0.0462 (15)	0.0416 (15)	0.0403 (15)	0.0016 (11)	0.0174 (12)	0.0002 (11)
C3	0.0378 (14)	0.0461 (15)	0.0366 (14)	0.0000 (10)	0.0136 (11)	−0.0002 (11)
C4	0.0364 (13)	0.0431 (15)	0.0348 (14)	−0.0009 (10)	0.0110 (11)	0.0019 (10)
C5	0.0420 (15)	0.0515 (17)	0.0360 (14)	−0.0011 (12)	0.0153 (12)	−0.0004 (12)
C6	0.0533 (17)	0.0416 (14)	0.0447 (16)	−0.0004 (12)	0.0212 (14)	0.0030 (12)

### Geometric parameters (Å, °)

**Table:**

S—C3	1.707 (3)	O2—H2A	0.8200
S—C2	1.712 (3)	C1—C2	1.501 (4)
N—C2	1.312 (3)	C1—H1A	0.9600
N—C4	1.371 (3)	C1—H1B	0.9600
F1—C6	1.320 (3)	C1—H1C	0.9600
F2—C6	1.321 (3)	C3—C4	1.371 (3)
F3—C6	1.319 (3)	C3—C5	1.496 (4)
O1—C5	1.185 (3)	C4—C6	1.501 (4)
O2—C5	1.317 (3)		
			
C3—S—C2	90.35 (12)	C5—C3—S	120.7 (2)
C2—N—C4	110.8 (2)	N—C4—C3	115.5 (3)
C5—O2—H2A	109.5	N—C4—C6	118.4 (2)
C2—C1—H1A	109.5	C3—C4—C6	126.1 (2)
C2—C1—H1B	109.5	O1—C5—O2	125.5 (2)
H1A—C1—H1B	109.5	O1—C5—C3	123.9 (2)
C2—C1—H1C	109.5	O2—C5—C3	110.6 (2)
H1A—C1—H1C	109.5	F3—C6—F1	105.6 (2)
H1B—C1—H1C	109.5	F3—C6—F2	107.0 (3)
N—C2—C1	124.3 (2)	F1—C6—F2	107.0 (3)
N—C2—S	114.24 (19)	F3—C6—C4	112.8 (2)
C1—C2—S	121.5 (2)	F1—C6—C4	112.4 (2)
C4—C3—C5	130.1 (3)	F2—C6—C4	111.5 (2)
C4—C3—S	109.18 (19)		
			
C4—N—C2—C1	178.7 (3)	S—C3—C4—C6	177.7 (2)
C4—N—C2—S	0.3 (3)	C4—C3—C5—O1	−14.8 (5)
C3—S—C2—N	−0.5 (2)	S—C3—C5—O1	162.6 (3)
C3—S—C2—C1	−179.0 (3)	C4—C3—C5—O2	166.9 (3)
C2—S—C3—C4	0.6 (2)	S—C3—C5—O2	−15.7 (3)
C2—S—C3—C5	−177.3 (2)	N—C4—C6—F3	−113.2 (3)
C2—N—C4—C3	0.1 (3)	C3—C4—C6—F3	68.6 (4)
C2—N—C4—C6	−178.2 (2)	N—C4—C6—F1	127.4 (3)
C5—C3—C4—N	177.1 (3)	C3—C4—C6—F1	−50.7 (4)
S—C3—C4—N	−0.5 (3)	N—C4—C6—F2	7.2 (4)
C5—C3—C4—C6	−4.7 (4)	C3—C4—C6—F2	−170.9 (3)

### Hydrogen-bond geometry (Å, °)

**Table:**

*D*—H···*A*	*D*—H	H···*A*	*D*···*A*	*D*—H···*A*
O2—H2A···N^i^	0.82	2.02	2.820 (3)	166
C1—H1A···O1^ii^	0.96	2.34	3.277 (4)	166

Symmetry codes: (i) *x*+1, −*y*+1/2, *z*−1/2; (ii) *x*−1, −*y*+1/2, *z*+1/2.
